# A Hybrid Model of Feature Extraction and Dimensionality Reduction Using ViT, PCA, and Random Forest for Multi-Classification of Brain Cancer

**DOI:** 10.3390/diagnostics15111392

**Published:** 2025-05-30

**Authors:** Hisham Allahem, Sameh Abd El-Ghany, A. A. Abd El-Aziz, Bader Aldughayfiq, Menwa Alshammeri, Malak Alamri

**Affiliations:** 1 Department of Information Systems, College of Computer and Information Sciences, Jouf University, Sakakah 42421, Saudi Arabia; allahem@ju.edu.sa (H.A.); saabdelwahab@ju.edu.sa (S.A.E.-G.); bmaldughayfiq@ju.edu.sa (B.A.); 2 Department of Computer Science, College of Computer and Information Sciences, Jouf University, Sakakah 42421, Saudi Arabia; mhalshammeri@ju.edu.sa (M.A.); mzalamri@ju.edu.sa (M.A.)

**Keywords:** brain tumor MRI dataset, brain tumors, cancer, deep learning, machine learning, MRI, PCA, RF, ViT

## Abstract

**Background/Objectives:** The brain serves as the central command center for the nervous system in the human body and is made up of nerve cells known as neurons. When these nerve cells grow rapidly and abnormally, it can lead to the development of a brain tumor. Brain tumors are severe conditions that can significantly reduce a person’s lifespan. Failure to detect or delayed diagnosis of brain tumors can have fatal consequences. Accurately identifying and classifying brain tumors poses a considerable challenge for medical professionals, especially in terms of diagnosing and treating them using medical imaging analysis. Errors in diagnosing brain tumors can significantly impact a person’s life expectancy. Magnetic Resonance Imaging (MRI) is highly effective in early detection, diagnosis, and classification of brain cancers due to its advanced imaging abilities for soft tissues. However, manual examination of brain MRI scans is prone to errors and heavily depends on radiologists’ experience and fatigue levels. Swift detection of brain tumors is crucial for ensuring patient safety. **Methods:** In recent years, computer-aided diagnosis (CAD) systems incorporating deep learning (DL) and machine learning (ML) technologies have gained popularity as they offer precise predictive outcomes based on MRI images using advanced computer vision techniques. This article introduces a novel hybrid CAD approach named ViT-PCA-RF, which integrates Vision Transformer (ViT) and Principal Component Analysis (PCA) with Random Forest (RF) for brain tumor classification, providing a new method in the field. ViT was employed for feature extraction, PCA for feature dimension reduction, and RF for brain tumor classification. The proposed ViT-PCA-RF model helps detect early brain tumors, enabling timely intervention, better patient outcomes, and streamlining the diagnostic process, reducing patient time and costs. Our research trained and tested on the Brain Tumor MRI (BTM) dataset for multi-classification of brain tumors. The BTM dataset was preprocessed using resizing and normalization methods to ensure consistent input. Subsequently, our innovative model was compared against traditional classifiers, showcasing impressive performance metrics. **Results:** It exhibited outstanding accuracy, specificity, precision, recall, and F1 score with rates of 99%, 99.4%, 98.1%, 98.1%, and 98.1%, respectively. **Conclusions**: Our innovative classifier’s evaluation underlined our model’s potential, which leverages ViT, PCA, and RF techniques, showing promise in the precise and effective detection of brain tumors.

## 1. Introduction

The brain, the most intricate organ in the human body, manages all functions and activities. It consists of numerous neurons—unique cells transmitting electrical and chemical messages; the brain is subdivided into various areas, each serving distinct roles such as thinking, processing senses, controlling movement, and managing emotions [1]. Neurons, also called nerve cells, are the essential building blocks of the brain and nervous system. They are specialized cells with a unique design for sending messages throughout the body. Each neuron comprises a cell body known as the soma, dendrites, and an axon. The dendrites receive signals from other neurons and send them towards the cell body.

On the other hand, the axon carries signals away from the cell body to other neurons or specific cells. Neurons communicate by transmitting electrical impulses called action potentials between each other. Upon stimulation, a neuron produces an action potential that travels along the axon to the axon terminals. At the terminals, it prompts the release of neurotransmitters, enabling communication between neurons [2].

The brain processes signals from the environment, enabling individuals to observe, comprehend, and engage with their surroundings, which involves focus, recollection, speech, problem solving, and decision making. The brain manages both deliberate and unconscious movements by coordinating muscles and nerves. People can walk, talk, hold objects, and execute various physical actions through motor control. Sensory information from the environment is received and deciphered by the brain through the senses, including vision, hearing, touch, taste, and smell. It merges and structures these data to form perceptions of the environment. The brain is crucial in feeling, expressing, and managing emotions. It encompasses intricate interactions among brain areas, like the limbic system and prefrontal cortex, impacting emotional reactions and conduct [1].

Brain tumors are abnormal cell growth that can occur in the brain or the central nervous system (CNS). They are categorized as either non-cancerous (benign) or cancerous (malignant). Benign tumors develop from cells in the brain or nearby CNS structures. They grow slowly, have clear borders, and are usually enclosed by a fibrous capsule. While not cancerous, they can still cause symptoms depending on their size, location, and proximity to vital brain structures. Common benign brain tumor types include meningiomas, pituitary adenomas, acoustic neuromas, craniopharyngiomas, and schwannomas. Symptoms vary based on the tumor’s location and size and may include headaches, seizures, cognitive changes, vision or hearing issues, motor problems, nausea, vomiting, and hormonal imbalances. Diagnosis typically involves imaging tests like MRI or (computed tomography) CT scans, neurological exams, and sometimes biopsy or surgical removal for analysis. Treatment options depend on tumor specifics and can include observation, surgery, radiation therapy, or stereotactic radiosurgery to manage symptoms and prevent complications. Overall, the outlook for benign brain tumors is positive compared to malignant ones. Surgical removal often leads to symptom resolution and long-term survival, although outcomes may vary based on tumor characteristics and treatment success [3,4,5].

Malignant brain tumors, also referred to as brain cancer or primary brain tumors, are irregular cell growths found in the brain or surrounding structures of the central nervous system (CNS) that display cancer-like qualities. Unlike benign tumors, malignant tumors lack defined borders, grow uncontrollably, invade nearby tissues, and have the potential to spread to other parts of the brain or CNS. A common type of malignant brain tumor is glioma. Gliomas originate from glial cells, which are supportive cells that surround and provide nutrients to neurons in the brain. These tumors can vary in aggressiveness and can occur in different parts of the brain, including the cerebral hemispheres, brainstem, and spinal cord. Gliomas are classified based on their specific cell type and location within the brain, with common subtypes including astrocytomas, oligodendrogliomas, and glioblastomas. Malignant brain tumor symptoms vary based on size, location, and growth rate. These symptoms may include headaches, seizures, cognitive changes, motor deficits, vision or hearing problems, nausea, vomiting, and mood swings. Diagnosis typically involves imaging tests like MRI or CT scans, neurological examinations, and sometimes biopsy or surgical removal of the tumor for analysis. Treatment for malignant brain tumors depends on factors such as tumor type, size, location, grade, and the patient’s overall health. Treatment strategies may involve surgery, radiation therapy, chemotherapy, targeted therapy, or immunotherapy. The main objective of treatment is to shrink or eradicate the tumor, alleviate symptoms, and prolong life. The outlook for recovery can differ based on various factors, including the type and grade of the tumor, its location, how much has been surgically removed, as well as the patient’s age and overall health condition. Malignant brain tumors generally have worse outcomes compared to benign tumors, with aggressive types like glioblastoma multiforme posing significant challenges in treatment [6,7].

Brain and CNS cancer rank among the top causes of mortality. Studies predict that nearly 18,990 individuals will be affected by CNS cancer in the upcoming year, with 11,020 cases in men and 7970 cases in women. Global data from 2020 show that 251,329 deaths occurred due to primary cancerous brain and CNS tumors [8]. The American Cancer Society anticipates approximately 3490 new cases of brain and CNS cancer in 2023, with 1900 cases in males and 1590 cases in females [9]. The survival rate for brain tumors ranges from 5 to 13 years and is approximately 33.4% per 100,000 individuals. In the year 2020, there were about 308,000 new cases of brain cancer, representing roughly 1.6% of all new cancer diagnoses. Brain tumors accounted for around 251,000 deaths, making up about 2.5% of all cancer-related fatalities. As of 2016, brain tumors surpassed leukemia as the primary cause of cancer-related death among children aged 0 to 14 in the United States [10]. Among adolescents and young adults aged 15 to 39, brain tumors rank as the third most common type of cancer [11]. Detecting these cancers early and accurately classifying them present significant challenges, yet they are vital. Ongoing efforts within the field of neurology aim to enhance detection and classification methods continually.

In the initial phases, signs of a brain tumor can be nonspecific and similar to those of other milder ailments. These signs might consist of head pain, alterations in eyesight, speech challenges, seizures, and alterations in emotions or actions. Consequently, they might not promptly arouse suspicion of a brain tumor [12]. Several imaging techniques are used for the detection and evaluation of brain tumors [1,13,14], such as MRI scans, CT scans, Positron Emission Tomography (PET) scans, CT Perfusion Imaging, MR Spectroscopy, Diffusion-Weighted Imaging (DWI), and Functional MRI (fMRI).

While imaging techniques such as MRI, CT scans, PET scans, and others are valuable tools for detecting and evaluating brain tumors, they also have certain limitations, such as false positives, false negatives, resolution limitations, tumor heterogeneity, the invasive nature of some techniques, and cost and accessibility.

Analyzing brain scans by considering the above aspects can be difficult and time-consuming for healthcare professionals such as clinicians, physicians, and radiologists. Radiologists can take advantage of autonomous computers with rapid processing capabilities [15,16]. Implementing CAD systems for brain tumor classification can streamline the process for doctors and radiologists and help them save valuable time [1].

Artificial intelligence (AI) describes how computers imitate human thought processes. These activities involve acquiring knowledge, applying rules, making decisions based on those rules, and adjusting actions accordingly. AI is used in different fields, such as healthcare, to help identify cancer. AI programs assess medical images like X-rays, CT scans, MRI scans, and mammograms to pinpoint questionable regions that could suggest the existence of tumors or irregularities [16,17]. CAD systems leverage DL and ML algorithms to assist radiologists in detecting and diagnosing cancers from medical imaging data. DL techniques are utilized to identify important aspects in medical images for describing tumors, such as their shape, texture, intensity, and spatial connections in the images. CNNs are frequently employed to automatically extract these features by recognizing layered patterns in the image data [18].

This research introduces a novel hybrid approach called the ViT-PCA-RF model for identifying brain tumors. The model utilizes ViT for feature extraction, PCA for reducing feature dimensions, and RF for categorizing brain tumors. This model aids healthcare professionals in the early detection of brain cancer. By streamlining diagnosis time and lowering related costs, it eases the workload on radiologists and provides them with an automated tool for accurately assessing brain function, ultimately reducing the risk of misdiagnosis. Testing was conducted on the BTM dataset to classify brain tumors into multiple categories. The dataset underwent preprocessing procedures such as resizing and normalization to ensure uniform input. It was split into 81.33% (5712 CT images) for the training set and 18.67% (1311 CT images) for the testing set. Comparative analysis with conventional classifiers highlighted the strong performance of our innovative model. The assessment of our new classifier emphasized the potential of our approach, which combines the ViT, PCA, and RF methods, demonstrating effectiveness in accurately detecting brain tumors. Below is an overview of the impacts our research has had.

- We created a new hybrid ViT-PCA-RF model that accurately detects brain cancer using ViT, PCA, and RF.
- We used ViT to extract features, PCA for reducing feature dimensions, and RF for classification of brain tumor.
- The enhanced hybrid model that combined ViT, PCA, and RF was carefully structured to maintain a good balance and prevent problems with overfitting.
- In a comparison with three hybrid ML models, our fine-tuned ViT-PCA-RF demonstrated exceptional effectiveness when handling precise multi-classification assignments.
- ViT showed outstanding performance in accurately extracting features compared to other DL models.
- Our new model showed outstanding precision when identifying brain cancer, requiring little time and effort.
- Our suggested model offers pathologists substantial advantages, allowing for early identification and proper management of brain cancer.
- The application of our highly optimized ViT-PCA-RF model in the early identification of cancer demonstrated potential as an essential asset in pathology, assisting in prompt and personalized patient treatment.
- An optimized hybrid model we designed delivered impressive results, boasting an accuracy rate of 99%, specificity of 99.4%, precision of 98.1%, recall of 98.1%, and an F1 score of 98.1%.

The structure of this paper is as follows: Section 2 delves into a review of brain diagnostic systems in the existing literature. In Section 3, we outline the preprocessing procedures for the BTM dataset and the methodology of our model. Section 4 showcases the experimental results of the proposed model. Finally, Section 5 offers the concluding remarks on our proposed model.

## 2. Literature Review

Brain tumor diagnosis is a popular area of research in analyzing medical images. Numerous studies approach this issue from various angles, resulting in a diverse range of investigations.

### 2.1. Transformer-Based Models

J. Wang et al. [19] proposed a novel approach named RanMerFormer for classifying brain tumors using a CAD model. The method involved leveraging a pre-trained ViT as the central model. Furthermore, they introduced a merging technique to remove unnecessary components in the ViT, significantly improving computational efficiency. To complete the RanMerFormer model, they incorporated a random vector functional-link mechanism, allowing for fast training. By conducting extensive simulations on two well-known benchmark datasets from Kaggle and Figshare websites, they demonstrated that RanMerFormer surpassed existing methods in brain tumor classification with an accuracy of 98.86%. The resulting RanMerFormer model displayed potential for practical use in diagnosing brain tumors.

### 2.2. CNN-Based Approaches

Z. Rasheed et al. [20] proposed a novel technique that combined image enhancement methods—specifically Gaussian-blur-based sharpening and Contrast Limited Adaptive Histogram Equalization (CLAHE). The primary aim of this approach was to effectively categorize various types of brain tumors, such as Glioma, Meningioma, and Pituitary tumors, as well as cases without tumors. The algorithm underwent extensive testing using the BTM dataset, and its results were compared to existing models, including VGG16, ResNet50, VGG19, InceptionV3, and MobileNetV2. The results of the experimental approach demonstrated an impressive classification accuracy of 97.84%, a precision rate of 97.85%, a recall rate of 97.85%, and an F1 score of 97.90%.

M. A. Gómez-Guzmán et al. [21] investigated seven different CNN models for categorizing brain tumors. One of these models was a simple CNN, while the remaining six were pre-trained CNN models. The dataset utilized in this study, known as BTM, consisted of three subsets: Figshare, SARTAJ, and Br35H, containing 7023 MRI images. These images were classified into four groups: Glioma, Meningioma, Pituitary tumors, and Normal brains. The models underwent training using these MRI images and various preprocessing methods. The CNN models studied included Generic CNN, ResNet50, InceptionV3, InceptionResNetV2, Xception, MobileNetV2, and EfficientNetB0. Among these models, InceptionV3 exhibited the highest performance, achieving an average accuracy of 97.12%.

K. Abdul Hannan et al. [22] introduced a unique method to categorize brain tumors into three main types, Glioma, Meningioma, and Pituitary, using a hierarchical DL technique. They utilized image fragments in a CNN to train the data and effectively classify them based on the different tumor types. This novel approach, called Hierarchical Deep Learning-Based Brain Tumor (HDL2BT) classification, used a CNN to identify and categorize brain tumors. The system classified tumors into four distinct types: Glioma, Meningioma, Pituitary, and No tumor. The model outperformed previous methods in brain tumor detection and segmentation with an accuracy rate of 92.13% and a miss rate of 7.87%.

A. Naseer et al. [23] focused on improving the early detection of brain tumors. They utilized CNN to enhance diagnostic precision. The research utilized the BR35H dataset, containing MRI scans of brain tumors, to train the CNN model. Researchers employed five diverse datasets to assess the model’s effectiveness and dependability—BMI-I, BMI-II, BMI-III, BTI, and BTS. By integrating various geometric data augmentation methods and statistical standardization, the CNN model was customized to process entirely new data, resulting in improved performance. The CNN-based CAD system introduced in the study outperformed other diagnostic techniques by achieving an average accuracy of around 98.8% and a specificity of about 99% in diagnosing brain tumors.

In their research, Saeedi et al. [24] designed and trained a convolutional auto-encoder network paired with a new 2D CNN. The architecture of the 2D CNN included several convolution layers, each with a 2 × 2 kernel function. The network consisted of eight convolutional layers and four pooling layers. Batch-normalization layers were inserted after each convolution layer. The altered auto-encoder network comprised a convolutional auto-encoder network linked with a convolutional classification network that utilized the output encoder layer from the former. To assess performance, the researchers contrasted six diverse ML techniques to classify brain tumors. The training accuracy reached 95.63% for the auto-encoder network and 96.47% for the 2D CNN. The average recall rates were 95% for the 2D CNN and 94% for the auto-encoder network. Both networks displayed strong performance, as indicated by the Receiver Operating Characteristic (ROC) curve showing an area under the curve of 0.99 or 1. Two ML techniques that stood out were K-nearest neighbors (KNN) and the multilayer perceptron (MLP), with KNN achieving an accuracy of 86% and MLP achieving 28%. The dataset utilized in the research consisted of 3264 T1-weighted contrast-enhanced MRI images, comprising images of healthy brains.

### 2.3. Hybrid Deep Learning and Machine Learning Models

Sarkar et al. [25] introduced a model that utilized explanations for DL to forecast different types of brain tumors, including Pituitary, Glioma, and Meningioma. They incorporated tools like Local Interpretable Model-agnostic Explanations (LIME), CNN, Shapley Additive Explanations (SHAP), and a dataset comprising MRI images. The model demonstrated an accuracy of 94.64%.

In a research study led by E. Mohammed Senan et al. [26], various analyses were undertaken to conclude cerebrum cancer by leveraging DL and ML techniques. The researchers employed ResNet-18 and AlexNet in conjunction with a Support Vector Machine (SVM) to predict outcomes related to brain tumors. The integration of ML and DL methods began with extracting features using AlexNet and ResNet-18, followed by classifying these features using SoftMax and SVM algorithms. Furthermore, the study utilized the average filter technique to enhance the quality of MRI images. The research findings indicated outstanding performance, achieving a sensitivity of 95.25%, accuracy of 95.10%, and specificity of 98.50%. The hybrid approach combining AlexNet and SVM emerged as the most effective methodology.

### 2.4. Reviews and Identified Challenges

Akinyelu et al. [27] conducted an in-depth analysis of brain tumor classification and segmentation techniques in a study. The researchers utilized ML, CNNs, capsule neural networks (CapsNets), and ViTs. Their research emphasized recent advancements in the field and the effectiveness of cutting-edge methods. Additionally, the team discussed critical challenges and unresolved issues, identified significant limitations, and highlighted areas that warrant further exploration.

Our research introduces a novel approach known as the ViT-PCA-RF model, which merges ViT, PCA, and RF techniques to detect brain tumors. ViT extracts features, PCA reduces their dimensions, and RF categorizes the tumors. Initially crafted for image classification, ViT has displayed promise in medical imaging, particularly in spotting brain tumors from MRI scans. By integrating self-attention mechanisms, ViT effectively captures long-distance relationships and spatial patterns within MRI images. This capability enables ViT to focus on relevant areas of the image, filtering out unnecessary background details and enhancing its precision in identifying subtle irregularities linked to brain tumors. Through a combination of self-attention layers and neural networks, ViT acquires detailed representations of input MRI images, facilitating accurate differentiation between normal brain tissue and tumor regions. Unlike traditional CNNs, ViT does not depend on fixed image patches or predefined spatial structures. Instead, it processes the entire MRI image as a sequence of patches, making it adaptable to various image resolutions and sizes without requiring extensive preprocessing.

PCA is a widely used technique for reducing data dimensions and extracting features in various applications, including analyzing MRI scans. One such important application is the detection of brain tumors in MRI images. PCA converts high-dimensional MRI data into a lower-dimensional format while retaining key information. This reduction aids in managing intricate datasets and simplifying tasks like distinguishing between tumor and healthy tissue regions. By streamlining MRI images, PCA addresses challenges linked to high dimensions and facilitates tasks such as categorizing different areas within the images. It concentrates on essential components that capture significant variations in pixel intensities, filtering out irrelevant details and noise. This noise elimination enhances image clarity, facilitating the identification of tumor-related patterns. In MRI image analysis, PCA identifies unique features based on their interrelationships, assisting in identifying variations in spatial layout and pixel intensities across different image areas. These features offer valuable insights into image data and support tasks like segmentation of tissue types, such as normal brain tissue and tumor regions.

## 3. Materials and Methods

### 3.1. Materials

**Figure 1:**
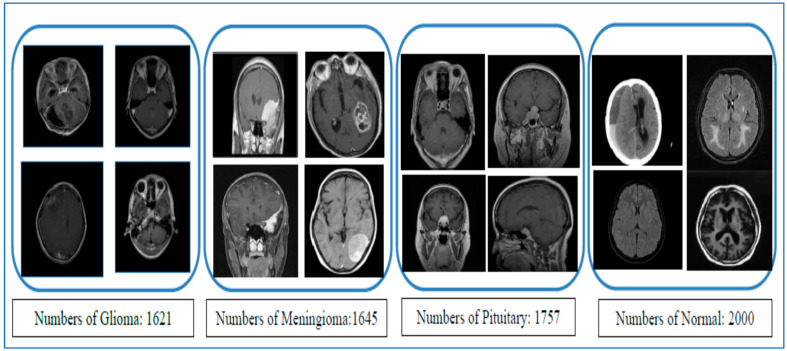
Samples of the BTM datasets.

**Figure 2:**
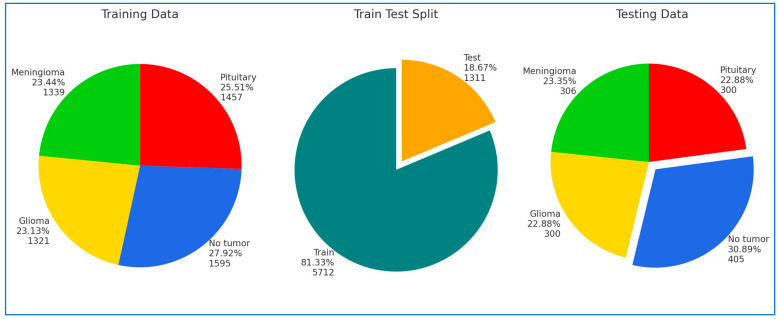
The classes’ distribution of the training and test datasets.

Our new ViT-PCA-RF model ViT-PCA-RF underwent evaluation using the BTM dataset, accessible on the Kaggle platform [28]. The BTM dataset comprises three primary datasets: Figshare [29], SARTAJ [30], and Br35H, utilized for images portraying individuals with healthy brains. This dataset features 7023 MRI images of the human brain, presented in grayscale and JPG formats, showcasing various types of brain tumors. The brain tumor images within the BTM dataset are divided into four categories: Normal (2000 images), Meningioma (benign) (1645 images), Pituitary (benign) (1757 images), and Glioma (malignant) (1621 images). Initially, to commence model training, the BTM dataset was divided into a training set comprising 81.33% of the total images (5712 MRI images) and a test set containing the remaining 18.67% (1311 MRI images). Sample images from the BTM dataset are displayed in Figure 1. The detailed breakdown of these classes within the BTM dataset, the training set, and the test set can be located in Table 1, Table 2 and Table 3, respectively. Furthermore, Figure 2 illustrates the class distribution in the training and test sets.

### 3.2. Methodology

**Figure 3:**
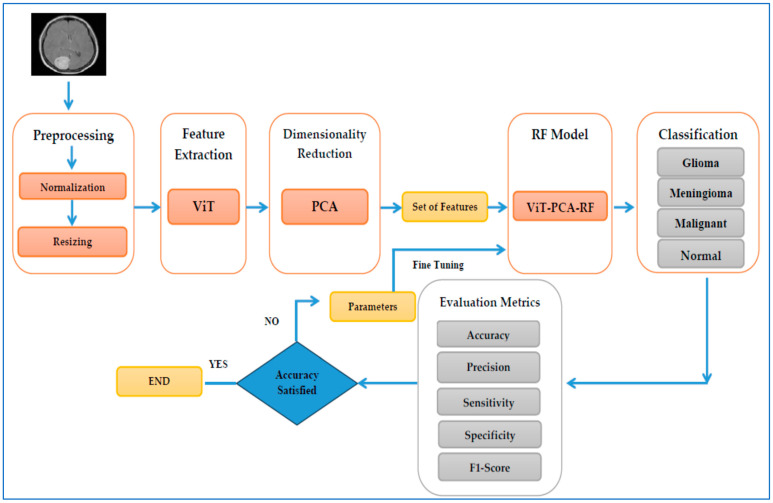
The proposed model architecture.

We have created a hybrid model, ViT-PCA-RF, to classify different types of brain tumors using multi-class classification. This model combines ViT for feature extraction, PCA for reducing dimensionality, and RF for classifying tumors. To evaluate its performance, we tested the model using the BTM dataset. The structure of the ViT-PCA-RF model is illustrated in Figure 3, and Algorithm 1 outlines the fine-tuning process of the three machine learning models. The key components of our proposed ViT-PCA-RF model include the following:

**Phase 1 (Datasets Preprocessing):** The BTM dataset was acquired from Kaggle and prepared in the initial phase. During the preparation process, the MRI images of the BTM dataset were adjusted in size and standardized [28].

**Phase 2 (Feature Extraction using ViT):** In phase two, we utilized ViT feature extraction to efficiently capture distant connections and spatial structures in MRI images. This function allows ViT to concentrate on significant image areas, removing irrelevant background information, and improving accuracy in detecting subtle abnormalities associated with brain tumors.

**Phase 3 (Dimensionality Reduction):** In the third phase, PCA decreased the complexity of high-dimensional MRI images, preserving a significant portion of the data variability. This simplification aids in streamlining the dataset and facilitating more straightforward analysis in later phases.

**Phase 4 (Datasets Splitting):** In the fourth phase, the BTM dataset was divided into a training set comprising 81.33% of the total images (5712 MRI images) and a test set containing the remaining 18.67% (1311 MRI images).

**Phase 5 (Train the Four ML Models):** In phase five, we trained four ML models on the training data from the BMT dataset. These models included Random Forest (RF), eXtreme Gradient Boosting (XGB), Decision Tree (DT), and Support Vector Machine (SVM). Furthermore, we fine-tuned their hyperparameters, such as the learning rate, batch size, and optimizer, to enhance their performance.

**Phase 6 (Fine-Tuning the Four Models):** In this phase, we fine-tuned their settings to improve their overall effectiveness.

**Phase 7 (Classification):** In the classification phase, we examined the four ML models using the test dataset to assess how well they can be applied broadly and to confirm their effectiveness with new data that have not been seen before.
**Algorithm 1:** The Proposed Fine-Tuned Hybrid ViT-PCA-RF Model1**Input** → BTM dataset BTM2**Output** ← Optimized Hybrid ViT-PCA-RF Model for Brain Cancer Diagnosis3**BEGIN**4 **STEP 1**: **Preprocessing of MRI images**5  **FOR EACH** MRI **IN** the BTM**DO**6   *Resize* MRI to 224 × 224.7  *Normalize* MRI pixel values from [0, 255] to [0, 1].8
  **END FOR**
9
 **STEP 2: Features Extraction**
10  *Apply* ViT for features extension.11
 **STEP 3: Dimensionality Reduction**
12  *Apply* PCA for dimensions reduction.13
 **STEP 4: BTM Splitting**
14  **SPLIT** BTM **INTO**15   *Train set*→81.33%.16   *Test set* →18.67%. 17
 **STEP 5: ML Models Training**
18  **FOR EACH** L IN [XGB, RF, SVM, DT] **DO**19   *Load* and *train* L on the train set.20
  **END FOR**
21
 **STEP 6: ML Models Fine Tuning**
22  **FOR EACH** Trained L IN [XGB, RF, SVM, DT] **DO**23   *Adjust* the hyperparameters of L to optimize its performance.24
  **END FOR**
25
 **STEP 7: Classification**
26  **FOR EACH** Optimized L IN [XGB, RF, SVM, DT] **DO**27    *Assess* the performance of L using the test dataset to gauge their ability to generalize.28
  **END FOR**
29**END**

#### 3.2.1. Dataset Preprocessing

Image enhancement for MRI scans involves various techniques to improve the quality of images before feeding them into computer vision systems. This preprocessing is crucial for optimizing the performance of CAD models. The key steps involved in preprocessing MRI images are as follows [31]:

- Resizing: Adjust image dimensions to a uniform size for consistency across the dataset. Models need to rely on images of the same size.
- Normalization: Normalize pixel values to a consistent range, such as [0, 1] or [−1, 1], to ensure stability during training and aid convergence.

In our research, the initial phase involved manipulating MRI scan data to enhance the performance of the ML models. One of the primary stages in this process was to standardize and adjust the size of the MRI images. Resizing and normalizing the MRI images were fundamental stages in preparing the data. Specifically, images from the BTM dataset were resized to dimensions of 224 × 224. Additionally, to ensure consistency, the MRI scans were adjusted from a range of 0 to 255 to a normalized scale of 0 to 1.

#### 3.2.2. Vision Transformer

**Figure 4:**
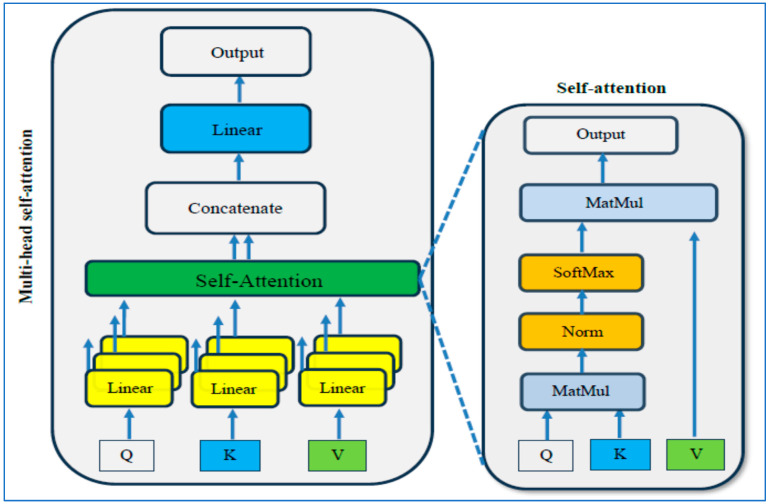
The self-attention process.

ViT is a type of advanced learning structure created by Dosovitskiy and colleagues in their research article “An Image is Worth 16 × 16 Words: Transformers for Image Recognition at Scale”. Unlike traditional CNNs, ViT uses the transformer design meant initially for natural language processing (NLP) in recognizing images. This inventive method employs self-attention mechanisms to study images, enabling the model to determine the significance of different input image areas during the forecasting stage. Self-attention is supported by an attention system that calculates weighted totals of input values depending on their relationships to specific inquiries or positions. The self-attention process is depicted in Figure 4 [32].

**Figure 5:**
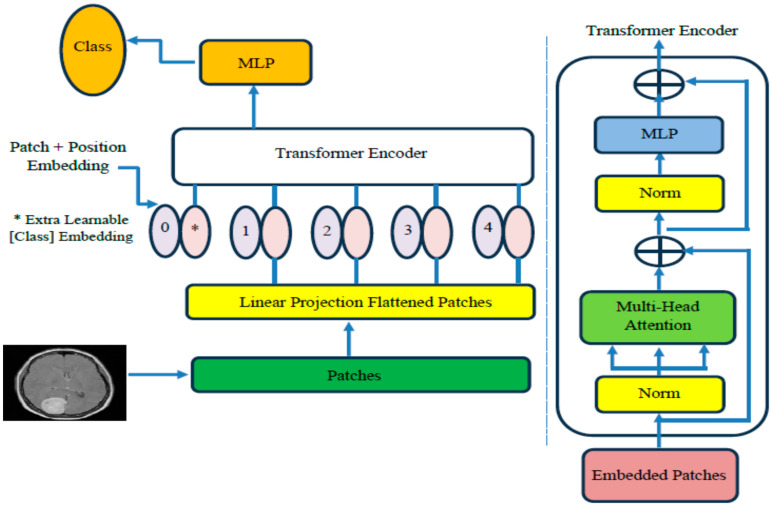
The ViT structure’s components.

The ViT structure’s components are shown in Figure 5 and itemized below [32]:

1. Patch Embeddings Process: The given image is divided into patches of a specific size, and each patch is transformed into a lower-dimensional embedding through a linear transformation. These embeddings, derived from the patches, serve as the input for the transformer encoder. The * represents the extra learnable [class] embedding, that is prepended to the sequence of image patch embeddings before being input into the Transformer Encoder. This token is used to aggregate global information from the entire image.
2. Transformer Encoder Description: The transformer encoder consists of numerous layers that incorporate self-attention mechanisms and MLP. The model can capture comprehensive contextual information from the entire image by processing the patch embeddings concurrently.
3. Positional Encoding Importance: Positional encodings are integrated into the patch embeddings to address the lack of inherent spatial understanding between patches in transformers. These encodings provide information about the relative positions of the patches within the image.
4. Utilization of Classification Head: Following the transformer encoder’s processing of the patch embeddings, a classification head is commonly included in the model. This component is utilized to make predictions for various tasks such as image classification, object detection, or segmentation.

The mathematical modeling for the ViT can be described as follows:

1. Input Embedding: Given an input image I, it is divided into fixed-size patches. Each patch $P_{i}$ is then linearly projected into a lower-dimensional embedding space using a learnable linear projection matrix $W_{e}$. Let $x_{i}$ represent the embedding of patch $P_{i}$, and then the input embeddings can be represented as follows:
  (1) $$x_{i}=W_{e}\;\cdot \;P_{i}$$
2. Transformer Encoder: The input embeddings and positional encodings are fed into a stack of transformer encoder layers. Each encoder layer consists of two main components:
  - Multi-Head Self-Attention: This mechanism allows the model to simultaneously attend different parts of the input embeddings. Given input embeddings X, the output of multi-head self-attention is calculated as follows:
    (2) $$Attension\left(Q,K,V\right)=softmax\left(\frac{QK^{T}}{\sqrt{d_{k}}}\right)\;V$$
    where $Q=XW_{Q},\;K=XW_{k},\;and\;V=XW_{v}$ are linear projections of the input embeddings, and $W_{Q},\;W_{k},\;and\;W_{v}$ are learnable linear projection matrices. $d_{k}$ represents the dimensionality of the key vectors.
  - Feedforward Neural Network: The output of the self-attention mechanism is then passed through a feedforward neural network (FFNN) consisting of two linear transformations separated by a non-linear activation function (typically ReLU). The output of the FFNN is calculated as follows:
    (3) $$FFNN\left(x\right)=ReLU\left(xW_{1}+b_{1}\right)W_{2}\;+b_{2}$$
    where $W_{1}$ and $W_{2\;}$ are learnable weight matrices and $b_{1}$ and $b_{2}$ are bias vectors.
3. Classification Head: The output embeddings from the transformer encoder are typically aggregated into a single representation (e.g., using average pooling), then passed through a linear layer followed by a softmax activation function to generate class probabilities for image classification tasks.

In the ViT-PCA-RF model with an input size of 224 × 224, an image initially arrives with 3 RGB channels and a resolution of 224 × 224 pixels. This model extracts 64 features from the image. The first step involves the patch layer, which divides the input scan into smaller sections known as “patches”. Each patch represents a unique portion of the image. The ViT structure modifies each patch, transforming image sections into a set of vector representations. These vector representations are then processed within the encoder. The encoder block receives data with dimensions of (None, 224, 224) and produces an output with the exact dimensions (None, 224, 224). Within the ViT model, the attention module aims to capture (974, 64) image features and highlight key elements. The encoder block enhances the features the attention mechanism identifies, refining them to be more detailed and comprehensive. Table 4 showcases the architecture of the ViT model used for feature extraction:

#### 3.2.3. PCA

PCA is a technique commonly used in ML and data analysis. Its main goal is to transform data with many dimensions into a space with fewer dimensions while preserving important information. PCA reduces the dimensions in a dataset by projecting it onto a lower-dimensional subspace. This is particularly useful for managing data with many dimensions, as it helps address the issues caused by having numerous dimensions and improves computational efficiency. PCA converts the original dimensions into a new set of linearly uncorrelated variables called principal components through an orthogonal transformation. These components are ordered based on the variance they explain, with the first component explaining the highest variance in the data, followed by the subsequent components. PCA aims to maximize the variance of the projected data along each principal component. PCA effectively identifies the main patterns or structures within the data by keeping components that capture the most variance. After computing the principal components, PCA allows for dimensionality reduction by selecting a subset of components that capture a significant portion of the total variance. The decision on which components to retain can be made based on criteria such as explained variance ratio or cumulative explained variance [33]. The mathematical modeling for the PCA can be described as follows:

If W represents the matrix containing the chosen principal components (with each column representing a principal component) and X represents the standardized data matrix, we can derive the dimensionality-reduced data $X_{reduced}$ by using the following formula:

(4) $$X_{reduced}=X\cdot \;W$$

PCA is a method utilized to simplify intricate MRI images. Initially, features were extracted using ViT. PCA then converted these features into a more straightforward form while preserving crucial information. These features were arranged in a matrix where each row corresponds to an MRI image, and each column represents a distinct feature from the image. PCA proceeded by identifying primary directions (principal components) in which the data exhibit the most variability. These components are blends of the initial features and were arranged based on the variation they encompass. Subsequently, a subset of these components was selected, capturing a significant portion of the data’s variance. The top k components were chosen according to the variance ratio, usually at around 98%. The selected components were then applied to transform the original feature matrix into a lower-dimensional space. This process effectively reduced the dimensionality of the feature space from 64 to 41 while retaining most of the critical information from the original MRI data.

#### 3.2.4. DT

A DT is a flexible supervised ML algorithm commonly used to categorize data. It divides the dataset into segments with similar characteristics, forming a tree structure. Each internal node in the structure decides based on a specific feature, while each leaf node represents a category label. The algorithm creates subsets of the data by splitting them based on feature values to create distinct segments related to the desired output. In essence, decision trees aim to replicate how humans make decisions hierarchically when problem solving. The algorithm categorizes instances or predicts numerical values by extracting if-else decision rules from the data. The algorithm determines the best feature to divide the data into more homogeneous subsets at each node, usually based on impurity or information gain measures. This process repeats until a specific stopping condition is met, like reaching a maximum tree depth or a minimum number of data points per leaf node [34].

The mathematical modeling for a DT is as follows:

Let us denote:

- DS as the dataset.
- *X* = {$x_{1,\;}x_{2}\ldots ,\;x_{n\;}$} represents the features of DS.
- Y is the target class.
- N is the current node in the DT.
- SC is the criteria splitting, which could be entropy or Gini index.
- $Split\;\left(DS,\;SC\right)$ as the function that chooses the most suitable way to split a dataset DS according to a specific criterion SC.

The DT model can be mathematically represented as a recursive function:

(5) $$Tree\;(N)=\left\{\begin{array}{c} Leaf\;\left(N\right),\;if\;stopping\;criteria\;is\;met\;\\ Split\;(DS,\;SC),\;\;\;\;\;\;\;\;\;\;\;\;\;\;\;\;\;\;\;\;\;\;\;\;\;\;\;\;\;\;\;Otherwise \end{array}\right.$$

When processing each non-terminal node, the function $Split\left(DS,SC\right)$ is responsible for choosing the feature and value combination that effectively partitions the dataset DS into smaller subsets. This selection reduces impurity or enhances information gain within the subsets.

#### 3.2.5. RF

RF is a flexible and robust data classification and prediction technique. It operates by training multiple DTs on various segments of the training dataset and then consolidating their predictions to generate a final prediction. In RF, the DTs are typically structured as binary trees. Each internal node signifies a decision based on a particular feature, whereas each leaf node corresponds to a category or group. The RF algorithm creates a collection of DTs by training each tree on a random subset of the training data, a process known as bagging. Furthermore, during each node split of the DT, only a random subset of features is considered for the division. This introduction of randomness and diversity within the group enhances its predictive accuracy. In RF, during the prediction phase, each DT in the combined model makes its prediction independently. For classification purposes, the final prediction for a data point is determined by the majority vote of the trees in the ensemble [35].

1. Training Phase:
  - Let us use X to represent the input feature matrix. This matrix has a size of m × n, where m stands for the number of samples and n is the number of features.
  - Let Y symbolize the matching desired vector of dimensions m × 1, used in a classification task. Let T_N be the number of DTs in the RF.
    - For each DT t_n=1,2,3,…T_N.
    - Randomly choose m’ training instances from the dataset with replacements to create a bootstrap sample.
    - Randomly choosing a collection of characteristics with k elements from the overall n features.
    - Train a DT on the bootstrap sample utilizing the chosen features.
2. Prediction Phase:
  - When a new sample input x is received, the input features are sent through each DT in the group.
  - The predicted class is determined by majority voting among the predictions of all DTs.

#### 3.2.6. XGB

XGB is a special type of ensemble learning method that falls under gradient boosting techniques. The main aim of XGB is to improve the speed and effectiveness of models. Essentially, XGB works by creating a series of basic models (often decision trees) in succession, which are then combined to form a more potent model. Developed using the programming language C++, XGB provides interfaces for various programming languages like Python, R, Java, and Julia. It utilizes L1 (Lasso) and L2 (Ridge) regularization techniques to prevent overfitting. Additionally, it includes functionalities to handle missing values in the dataset. During the training process, XGB autonomously learns how to deal with missing data, eliminating manual data preprocessing requirements. Furthermore, XGB uses tree-pruning methods to control the depth of individual trees, thereby reducing computational complexity and addressing overfitting.

XGB constructs DTs by iteratively making decisions and dividing the data based on optimal criteria to improve predictive accuracy. It looks for ideal points to split the data to refine their predictions, considering factors like information gain and purity. By combining numerous smaller, less accurate DTs, XGB creates a robust predictive model. It consistently enhances its performance by learning from prediction errors. Through intelligent strategies, XGB effectively improves its predictive accuracy by efficiently organizing data. Finally, it combines the predictions from all the decision trees to produce a final prediction [36].

#### 3.2.7. SVM

SVM is a specific supervised ML technique used to classify items into various categories. The fundamental concept of SVM involves determining the optimal hyperplane (a specialized line) that accurately segregates different clusters based on a set of characteristics. This optimal hyperplane, known as the margin, is designed to maximize the distance between these clusters. The margin represents the gap between the hyperplane and the nearest points from each cluster, referred to as support vectors. The primary objective of SVM is to establish a hyperplane that maximizes this margin, serving as the most effective line for predicting new, unseen data. When data cannot be separated using a linear hyperplane, SVM employs a kernel function to transform the feature space into a higher dimension. This transformation enables SVM to create a linear boundary in this new space, effectively distinguishing clusters that cannot be separated linearly [37].

In SVM, a decision boundary called a hyperplane is defined mathematically. This hyperplane separates the classes in the feature space. Here is a simple explanation of the mathematical formula employed in SVM:

Given a training dataset consisting of *n* samples $\left\{\left(x_{1,\;}y_{1}\right),\ldots ,\;\left(x_{n,\;}y_{n}\right)\right\}$, where $x_{i}$ is ith sample’s feature vector and $y_{i}$ is its class label $y_{i}\;\in \;\left\{C_{1},\;C_{2},\;\ldots ,\;C_{k}\right\}$, SVM seeks to identify the perfect line that effectively divides the data into two groups. In mathematical terms, this line can be defined by the equation so that it is articulated distinctly:

(6) $$w^{T}\;x+b=0$$

where w is the weight vector perpendicular to the hyperplane, x is the input feature vector, and b is the bias term.

SVM seeks to increase the space between the hyperplane and the closest data points from each category, known as the margin. This margin is defined as follows:

(7) $$margain=\frac{2}{\left\|w\right\|}$$

In cases where the data are not linearly separable, SVM allows for a soft margin, which permits some misclassification errors. This is achieved by introducing slack variables $\xi_{i\;}$ for each sample, allowing points to be on the wrong side of the margin or hyperplane. The objective is to minimize as follows:

(8) $$\frac{1}{2}\;\;{\left\|w\right\|}^{2\;}+C\sum_{i}^{n}\xi_{i\;}$$

Subjective to $y_{i}{\;(w}^{T}\;x+b$) $\geq 1-\xi_{i\;}$ for all I, where C is the regularization parameter, manages the balance between increasing the margin and decreasing the classification error.

## 4. Results and Discussion

### 4.1. Measured Performance Metrics

The effectiveness of ViT, VGG16, ResNet 50, DenseNet201, and Xception when used with DT, RF, XGB, and SVM is evaluated using equations from (9) to (15):

(9) $$Accuracy=\frac{\left(TP+TN\right)}{\left(TP+FP+TN+FN\right)}\;\;$$

(10) $$Precision=\frac{TP}{\left(TP+FP\right)}\;$$

(11) $$Sensivity=\frac{TP}{\left(TP+FN\right)}$$

(12) $$Specifity=\frac{TN}{\left(TN+FP\right)}$$

(13) $$F1\;score=\frac{2\times Precision\times Recall}{Precision+Recall}\;$$

(14) $$False\;Negative\;Rate\;(FNR)=\frac{FN}{TP+FN}\;$$

(15) $$Negative\;Predictive\;Value\;(NPV)=\frac{TN}{TN+FN}$$

**True Positive** (TP): When a classifier correctly predicts a positive outcome in classification tasks, it is known as a true positive.

**False Positive** (FP): Occurs when the classifier incorrectly predicts the presence of the positive class.

**True Negative** (TN): Occurs when the classifier correctly predicts the lack of the positive class.

**False Negative** (FN) refers to situations in classification tasks where the classifier wrongly predicts the absence of the positive class.

**Accuracy** is a metric used to evaluate the performance of a classification model. It represents the percentage of correctly classified instances out of all instances evaluated. This metric is dependable when the categories in the dataset are evenly distributed with similar numbers of instances. However, in situations with imbalanced datasets, where one category is much more prevalent than the others, accuracy may not be the most appropriate measure to rely on.

**Precision** measures the ratio of correctly identified TP out of all instances predicted as positive. When assessing a model, precision emphasizes its accuracy in making positive predictions. A high precision suggests that the model produces few false positive errors, indicating that its positive predictions are more likely to be correct.

**Sensitivity**, alternatively known as recall or true positive rate (TPR), is a measure used to assess the performance of a classification model. It indicates the percentage of true positive instances correctly identified by the model among all actual positive cases. Sensitivity portrays the model’s effectiveness in identifying positive cases within the dataset. A high sensitivity level suggests that the model minimizes the incorrect omission of positive instances, ultimately successfully capturing most positive cases.

The **F1 score** is a popular way to assess how well a classification model works. It considers both precision and sensitivity to thoroughly evaluate the model’s accuracy. The score can range between 0 and 1, where a higher F1 score shows that the model performs better. This measure is especially handy when dealing with datasets with unequal amounts of positive and negative instances.

### 4.2. Computational Requirements

In this research, we carried out four experiments on the public brain tumor dataset within the Kaggle environment. This environment was powered by an i7-10510U CPU running at 1.8 GHz and had 8 GB of RAM. We utilized Python version 3 and the TensorFlow library, a well-known DL framework developed by Google. The hyperparameters used in the two experiments, along with their specific values and processing times, are detailed in Table 5, Table 6 and Table 7.

### 4.3. The ViT-PCA-RF Model Assessment

In our research, we conducted four experiments on the Kaggle platform utilizing BTM and Figshare datasets. For the training phase, we partitioned the BTM dataset into 81.33% of the total images, amounting to 5712 MRI images. The remaining 18.67%, consisting of 1311 MRI images, was designated as the test set. The Figshare dataset was divided into 70% for training, 15% for testing, and 15% for validation.

The first experiment compared the ViT-PCA-RF model against three other hybrid machine learning models: ViT-PCA-DT, ViT-PCA-XGB, and ViT-PCA-SVM. In the second experiment, we evaluated ViT’s feature extraction capabilities in comparison to VGG16, ResNet50, DenseNet201, and Xception. The third and fourth experiments involved external validation of the ViT-PCA-RF model using an external dataset from Figshare. Following each experiment, the identified metrics (Equations (9)–(15)) were employed to assess the performance of the hybrid models.

**Our first experiment** categorized brain tumors into multiple classes using the test set of the BTM dataset. This involved utilizing ViT for feature extraction, PCA to adjust feature dimensions, and RF, DT, XGB, and SVM for classification purposes. The primary goal of this experiment was to distinguish between different types of brain tumors, leading to improved patient outcomes and a more efficient diagnostic process, ultimately reducing both time and costs for patients. The BTM dataset comprises four main classes: Normal, Meningioma, Pituitary, and Glioma. The outcomes of the ViT-PCA-RF, ViT-PCA-DT, ViT-PCA-XGB, and ViT-PCA-SVM hybrid models are detailed in Table 8, respectively. These tables present the average evaluation metrics for the four hybrid models used in the multi-classification task over the BTM dataset’s test set. The average accuracies achieved were 99%, 98.2%, 98.9%, and 98.8% for ViT-PCA-RF, ViT-PCA-DT, ViT-PCA-XGB, and ViT-PCA-SVM, respectively. Thus, the model ViT-PCA-RF demonstrated the highest level of accuracy.

ViT-PCA-DT achieved an average of 98.9% specificity, 3.8% FNR, 98.9% NPV, 96.5% precision, 96.5% recall, and 96.5% F1 score. ViT-PCA-XGB achieved the following average scores: 99.2% for specificity, 2.5% for FNR, 99.3% for NPV, 97.7% for precision, 97.7% for recall, and 97.7% for F1 score. ViT-PCA-SVM achieved average scores of 99.2% for specificity, 2.5% for FNR, 99.2% for NPV, 97.6% for precision, 97.6% for recall, and 97.6% for F1 score.

Therefore, ViT-PCA-RF demonstrated outstanding performance across various metrics. It achieved the highest averages for accuracy, specificity, NPV, precision, recall, and F1 score, at 99.4%, 99.4%, 98.1%, 98.1%, and 98.1%, respectively. Additionally, it boasted the lowest average FNR at 2.1%. This signifies that ViT-PCA-RF excels in multi-classification tasks, showcasing strong performance across various evaluation criteria.

The test set of the BTM dataset was divided into four main groups: Glioma, Meningioma, Normal, and Pituitary. We evaluated the effectiveness of four new hybrid models—ViT-PCA-RF, ViT-PCA-DT, ViT-PCA-XGB, and ViT-PCA—using a range of assessment measures such as accuracy, specificity, FNR, NPV, precision, recall, and F1 score for each category as shown in Table 9, Table 10, Table 11 and Table 12.

Among the different models assessed, ViT_PCA_RF stood out in the **Glioma** category, achieving exceptional results with the highest accuracy, specificity, precision, and F1 score, at 98.47%, 99.60%, 98.61%, and 96.60%, respectively. On the other hand, ViT-PCA-SVM demonstrated the highest NPV and recall, at 98.7% and 95.7%, while also exhibiting the lowest FNR of 4.3%.

In class **Meningioma**, ViT_PCA_RF achieved the highest performance metrics with the following scores: accuracy: 98.55%, specificity: 98.81%, NPV: 99.30%, precision: 96.14%, recall: 97.71%, F1 score: 96.92%. Furthermore, it demonstrated the lowest FNR at 2.29%.

In **the Normal** class, the ViT-PCA-RF model showed the most substantial performance metrics, boasting the highest accuracy, specificity, precision, and F1 score rates of 99.85%, 99.78%, 99.51%, and 99.75%, respectively. Furthermore, within the ViT-PCA-RF, ViT-PCA-DT, and ViT-PCA-XGB models, the NPV and recall rates reached 100%, while also showcasing the lowest false negative rate of 0%.

In the **Pituitary** class, ViT-PCA-SVM achieved the highest F1 score accuracy, specificity, precision, and F1 score scores, with 99.5%, 99.5%, 98.3%, and 98.8%, respectively. ViT-PCA-SVM and ViT-PCA-RF both reached the greatest NPV at 99.8%. Additionally, ViT-PCA-RF obtained the top overall score at 99.33%. Moreover, ViT-PCA-RF showcased the best recall rate at 99.33% and achieved the lowest FNR at 0.67%.

**Figure 6:**
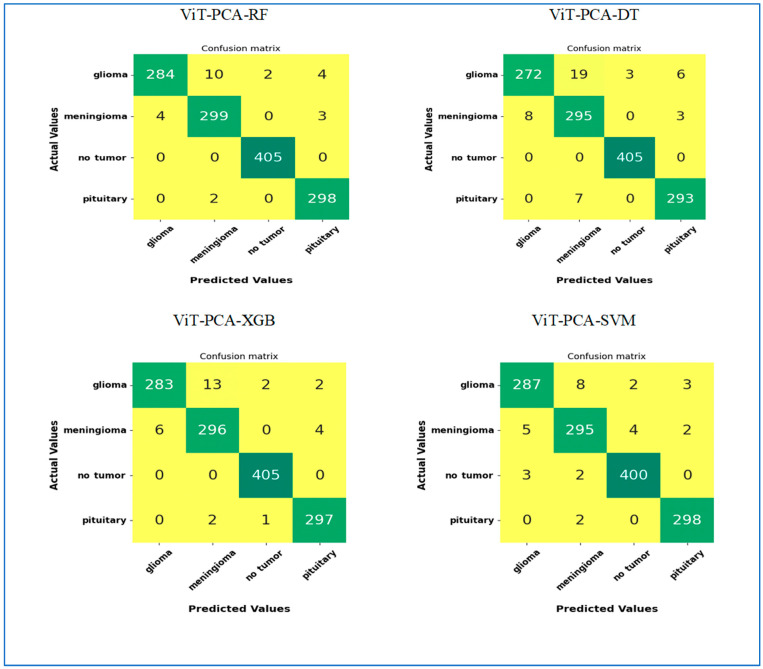
The confusion matrices of the four hybrid models for the multi-classification.

Figure 6 illustrates the performance results of the hybrid models ViT-PCA-RF, ViT-PCA-DT, ViT-PCA-XGB, and ViT-PCA-SVM when evaluated using the test set of the BTM dataset across four distinct classes: Glioma, Meningioma, Normal, and Pituitary. Within the BTM test set, there are 300 MRI scans for Glioma, 306 for Meningioma, 405 for Normal, and 300 for Pituitary.

In classifying MRI images related to **Glioma**, the hybrid ViT-PCA models yielded the following results: ViT-PCA-RF successfully classified 284 out of 300 MRI images, achieving an accuracy rate of 94.6% for the Glioma class. ViT-PCA-DT accurately classified 272 MRI images, with an accuracy rate of 90.6% for the Glioma class. ViT-PCA-XGB correctly classified 283 MRI scans, attaining an accuracy of 94.3%, specifically for the Glioma class. ViT-PCA-SVM demonstrated the strongest performance by correctly classifying 287 MRI scans, resulting in an accuracy rate of 95.6% for the Glioma class. Consequently, the models ranked based on their performance in the Glioma class are as follows: ViT-PCA-SVM, ViT-PCA-RF, ViT-PCA-XGB, and ViT-PCA-DT.

ViT-PCA-RF effectively classified 299 out of 306 MRI images, resulting in a 97.7% accuracy for the **Meningioma** category. ViT-PCA-DT and ViT-PCA-SVM accurately identified 295 MRI images, achieving a 96.4% accuracy rate for the Meningioma class. ViT-PCA-XGB accurately categorized 296 MRI scans, with a specific accuracy of 96.7% for the Meningioma class. The models ranked by their performance in the Meningioma class are as follows, from best to least performing: ViT-PCA-RF, ViT-PCA-XGB, ViT-PCA-SVM, and ViT-PCA-DT.

The hybrid models ViT-PCA-RF, ViT-PCA-XGB, and ViT-PCA-DT successfully categorized all 405 MRI images, resulting in a 100% accuracy rate for the **Normal** category. Among these models, ViT-PCA-SVM accurately identified 400 MRI images, achieving a 98.7% accuracy rate specifically for the Normal category. Therefore, when considering performance within the Normal class, the models can be ranked as ViT-PCA-RF, ViT-PCA-XGB, ViT-PCA-DT, and ViT-PCA-SVM.

ViT-PCA-RF and ViT-PCA-SVM effectively classified 298 out of 300 MRI images, resulting in an accuracy rate of 99.3% for the **Pituitary** category. ViT-PCA-DT correctly identified 293 MRI images, achieving a 97.6% accuracy for the Pituitary class. Additionally, ViT-PCA-XGB successfully categorized 297 MRI scans, with a notable accuracy of 99% for the Pituitary category. When considering the models’ performance in the Pituitary class, they can be ranked from best to least performing as follows: ViT-PCA-RF, ViT-PCA-SVM, ViT-PCA-XGB, and ViT-PCA-DT.

**In our second experiment,** we categorized brain tumors using the BTM dataset with the assistance of five DL models, ViT, VGG16, ResNet50, DenseNet201, and Xception, for feature extraction. Subsequently, we utilized PCA to reduce the features’ dimensions and applied the RF algorithm for classification. The primary aim of this experiment was to assess the feature extraction effectiveness of ViT compared to VGG16, ResNet50, DenseNet201, and Xception. The BTM dataset comprised four key classes: Normal, Meningioma, Pituitary, and Glioma. Results from our second experiment, including hybrid models like ViT-PCA-RF, VGG16-PCA-RF, ResNet50-PCA-RF, DenseNet201-PCA-RF, and Xception-PCA-RF, are detailed in Table 13. These tables demonstrate the average performance metrics of the five hybrid models in the multi-classification task on the test set of the BTM dataset. The average accuracies obtained were 99% for ViT-PCA-RF, 97% for VGG16-PCA-RF, 95.7% for ResNet50-PCA-RF, 96.2% for DenseNet201-PCA-RF, and 95.3% for Xception-PCA-RF. Consequently, the ViT-PCA-RF model displayed the highest accuracy level among all the models.

VGG16-PCA-RF recorded an average of 98% specificity, 6.5% FNR, 98.1% NPV, 94% precision, 94% recall, and 93.9% F1 score. ResNet50-PCA-RF recorded the following average scores: 97.2% for specificity, 9.4% for FNR, 97.2% for NPV, 91.2% for precision, 91.3% for recall, and 91.2% for F1 score. DenseNet201-PCA-RF recorded average scores of 97.5% for specificity, 8.3% for FNR, 97.6% for NPV, 93.0% for precision, 92.4% for recall, and 92.3% for F1 score. Xception-PCA-RF recorded the following average scores: 96.9% for specificity, 10.2% for FNR, 97% for NPV, 90.6% for precision, 90.5% for recall, and 90.5% for F1 score.

Therefore, ViT-PCA-RF showed excellent performance in different areas. It had the highest average scores for accuracy, specificity, NPV, precision, recall, and F1 score, at 99%, 99.4%, 99.4%, 98.1%, 98.1%, and 98.1%, respectively. Additionally, it had the lowest average FNR at 2.1%. This indicates that ViT-PCA-RF is very good at handling tasks involving multiple categories and performs well across various evaluation standards.

The test set of the BTM dataset was categorized into four primary groups: Glioma, Meningioma, Normal, and Pituitary. We assessed the performance of four novel hybrid models—ViT-PCA-RF, VGG16-PCA-RF, ResNet50-PCA-RF, DenseNet201-PCA-RF, and Xception-PCA-RF—by measuring various evaluation metrics, including accuracy, specificity, FNR, NPV, precision, recall, and F1 score for every classification as shown in Table 9 and Table 14, Table 15, Table 16, Table 17.

**In the Glioma class**, ViT_PCA_RF stood out among all the models assessed for its exceptional performance. It delivered impressive outcomes in accuracy, specificity, NPV, precision, recall, and F1 score, achieving scores of 98.47%, 99.60%, 98.44, 98.61%, 94.67, and 96.60%. Notably, ViT-PCA-RF exhibited the lowest FNR at 5.33%.

**In the class Meningioma,** the ViT_PCA_RF model showed outstanding performance with the following results: accuracy: 98.55%, specificity: 98.81%, NPV: 99.30%, precision: 96.14%, recall: 97.71%, F1 score: 96.92%. Moreover, it exhibited the smallest FNR at 2.29%.

**In the Normal class**, the DenseNet201-PCA-RF model displayed outstanding performance metrics. It achieved the highest accuracy, specificity, precision, and F1 score rates, all at 99.9% or 100%. Additionally, among the models ViT-PCA-RF, VGG16-PCA-RF, and ResNet50-PCA-RF, both the NPV and recall rates achieved 100%, along with maintaining the lowest FNR of 0%.

**In the Pituitary class,** ViT-PCA-RF achieved top scores in accuracy, specificity, NPV, precision, recall, and F1 score, with 99.31%, 99.31%, 99.80%, 97.70%, 99.33%, and 98.51%, respectively. Furthermore, ViT-PCA-RF attained the lowest FNR at 0.67%.

**Figure 7:**
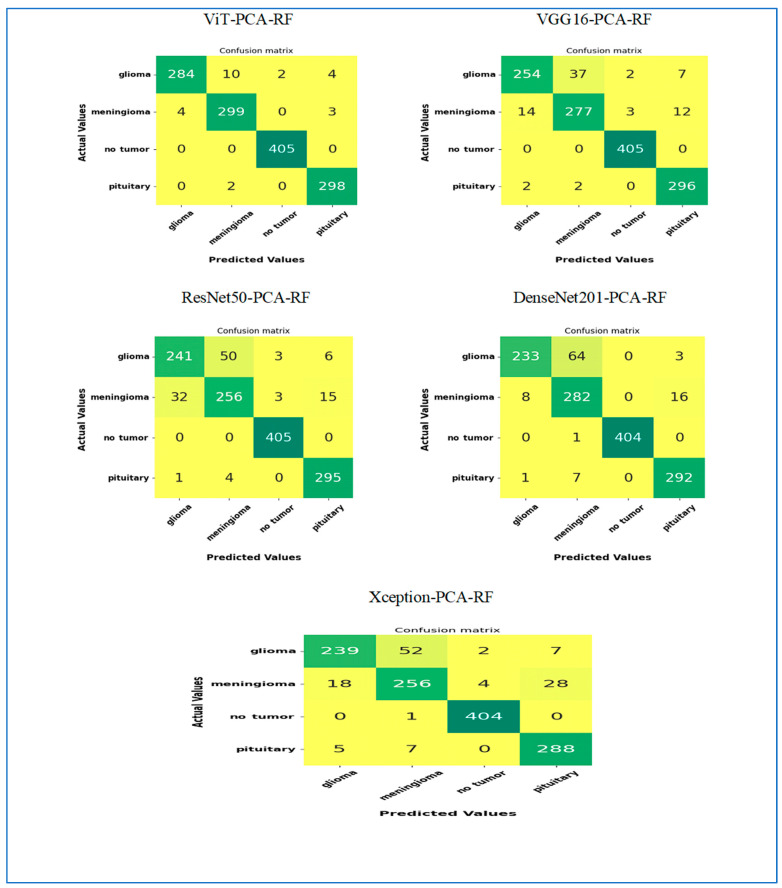
The confusion matrices of the five hybrid models for the multi-classification.

Figure 7 illustrates the performance results of the hybrid models ViT-PCA-RF, VGG16-PCA-DT, ResNet50-PCA-RF, DenseNet201-PCA-RF, and Xception-PCA-RF when evaluated using the BTM dataset across four distinct classes: Glioma, Meningioma, Normal, and Pituitary. Within the BTM test set, there are 300 MRI scans for Glioma, 306 for Meningioma, 405 for Normal, and 300 for Pituitary.

In classifying MRI images of the class **Glioma**, the hybrid ViT-PCA-RF accurately classified 284 out of 300 MRI images, achieving a 94.6% accuracy rate for Glioma. VGG16-PCA-RF achieved an 84.7% accuracy in classifying 254 MRI images related to Glioma. ResNet50-PCA-RF correctly classified 241 MRI scans with an accuracy rate of 80.3% for the Glioma category. DenseNet201-PCA-RF demonstrated the strongest performance by accurately classifying 233 MRI scans, resulting in a 77.6% accuracy for Glioma. Xception-PCA-RF achieved a 79.6% accuracy in classifying 239 MRI images specifically for Glioma. The models’ rank in terms of Glioma classification performance is as follows: ViT-PCA-RF, VGG16-PCA-RF, ResNet50-PCA-RF, Xception-PCA-RF, and DenseNet201-PCA-RF.

In classifying MRI images of the class **Meningioma**, the hybrid ViT-PCA-RF accurately classified 299 out of 306 MRI images, achieving a 97.7% accuracy rate for Meningioma. VGG16-PCA-RF achieved a 90.5% accuracy in classifying 277 MRI images related to Meningioma. ResNet50-PCA-RF and Xception-PCA-RF correctly classified 256 MRI scans with an accuracy rate of 83.6% for the Meningioma category. DenseNet201-PCA-RF demonstrated the strongest performance by accurately classifying 282 MRI scans, resulting in a 92.1% accuracy for Meningioma. The models’ rank in terms of Meningioma classification performance is ViT-PCA-RF, DenseNet201-PCA-RF, VGG16-PCA-RF, ResNet50-PCA-RF, and Xception-PCA-RF.

In the classification of MRI images of the class **Normal**, the hybrid models ViT-PCA-RF, VGG16-PCA-RF, and ResNet50-PCA-RF all achieved a perfect classification, accurately identifying all 405 MRI images in the Normal class, resulting in a 100% accuracy rate. DenseNet201-PCA-RF and Xception-PCA-RF also performed exceptionally well, correctly classifying 404 MRI scans, leading to a 99.75% accuracy rate for the Normal class. Ranking of models based on their performance in Normal classification: ViT-PCA-RF, VGG16-PCA-RF, ResNet50-PCA-RF, Xception-PCA-RF, and DenseNet201-PCA-RF.

In the classification of MRI images of the class **Pituitary**, the following models achieved the following accuracy rates: ViT-PCA-RF: 99.3% accuracy (298 out of 300 images), VGG16-PCA-RF: 98.6% accuracy (296 out of 300 images), ResNet50-PCA-RF: 98.3% accuracy (295 out of 300 images), DenseNet201-PCA-RF: 97.3% accuracy (292 out of 300 images), and Xception-PCA-RF: 96% accuracy (288 out of 300 images). Rank of models based on Pituitary classification performance: ViT-PCA-RF, VGG16-PCA-RF, ResNet50-PCA-RF, DenseNet201-PCA-RF, and Xception-PCA-RF.

### 4.4. External Validationof the ViT-PCA-RF Model

**In the third experiment,** we conducted external validation of the ViT-PCA-RF model using the external dataset from Figshare. This brain tumor dataset contains 3064 T1-weighted contrast-enhanced images from 233 patients, featuring three types of brain tumors: meningioma (708 slices), glioma (1426 slices), and pituitary tumor (930 slices). The dataset was divided into 70% for training, 15% for testing, and 15% for validation. The testing set has three brain tumors: meningioma, glioma, and pituitary tumor.

In the third experiment, we utilized ViT for feature extraction, applied PCA to modify feature dimensions, and employed RF, DT, XGB, and SVM for classification. The main objective of this experiment was to externally validate the combination of ViT, PCA, and RF.

The results of the hybrid models ViT-PCA-RF, ViT-PCA-DT, ViT-PCA-XGB, and ViT-PCA-SVM are summarized in Table 18. Table 18 presents the average evaluation metrics for the four hybrid models applied to the multi-classification task, using the test set from the Figshare dataset. The average accuracies obtained were 95.85% for ViT-PCA-RF, 95.36% for ViT-PCA-DT, 95.36% for ViT-PCA-XGB, and 95.36% for ViT-PCA-SVM. Therefore, the ViT-PCA-RF model recorded the highest accuracy.

The performance of five models—ViT-PCA-RF, ViT-PCA-DT, ViT-PCA-XGB, ViT-PCA-SVM, and A-SVM—was assessed using various metrics: accuracy, specificity, false FNR, NPV, precision, recall, and F1 score.

The ViT-PCA-RF model exhibited the highest specificity (96.54%) and NPV (97.05%), showcasing its strong capability to accurately identify negative cases while minimizing false positives. Its precision was 93.94%, and recall stood at 91.90%, leading to the highest F1 score of 92.74% among the models tested. Additionally, it recorded the lowest FNR at 8.10%, indicating fewer false negatives than the other models.

The ViT-PCA-DT model showed specificity at 96.44% and an NPV of 96.47%. Its precision (91.96%) and recall (91.81%) were slightly lower than those of ViT-PCA-RF, resulting in an F1 score of 91.88%. The FNR for this model was 8.19%, which was marginally higher than that of ViT-PCA-RF, suggesting a slightly greater number of false negatives.

Similarly, the ViT-PCA-XGB model had slightly lower specificity (96.17%) and a higher FNR (8.82%) compared to ViT-PCA-DT. Its NPV was 96.62%. While it achieved a precision of 92.91%, its recall of 91.18% was lower, leading to an F1 score of 91.89%.

In contrast, the ViT-PCA-SVM model had significantly lower specificity (64.24%) and NPV (64.99%) compared to the other models. It recorded a substantially lower FNR of 2.49%, indicating better identification of positive cases. However, its precision (62.57%) and recall (64.51%) were considerably lower, resulting in the lowest F1 score of 63.50% among the models.

In summary, the ViT-PCA-RF model exhibited superior performance across most metrics, particularly in specificity, precision, recall, and F1 score. On the other hand, the ViT-PCA-SVM model fell short in precision, specificity, and F1 score, despite its low false negative rate.

Table 19, Table 20, Table 21 and Table 22 present the class-wise results of the four hybrid models. **The ViT-PCA-DT model** demonstrated strong classification performance across three types of brain tumors: Glioma, Meningioma, and Pituitary tumors. **For the Glioma class**, the model achieved an accuracy of 95.52%, with a specificity of 95.65%. The FNR was 4.62%, and the NPV was also 95.65%. Precision, recall, and F1 score were consistently high at 95.38%, indicating a balanced performance in identifying Glioma cases. **For the Meningioma class**, the model reached an accuracy of 94.03%, but the specificity was notably lower at 96.51%, indicating a high false positive rate. Nevertheless, the FNR remained low at 14.94%, and the NPV was 95.90%. Precision was recorded at 87.06%, while recall was 85.06%, resulting in an F1 score of 86.05%. The low specificity highlighted the model’s difficulty in accurately identifying non-Meningioma cases. **For the Pituitary class**, the model performed best in the Pituitary class, achieving an accuracy of 96.52% and a specificity of 97.16%. The FNR was 5.00%, and the NPV reached 97.86%. Precision was 93.44%, and recall was 95.00%, leading to an F1 score of 94.21%, indicating robust and balanced performance.

The **ViT-PCA-SVM** demonstrated strong performance across most evaluation metrics, with some variation depending on the tumor type. **For Glioma**, the model achieved an accuracy of 95.02%, a specificity of 95.65%, and an FNR of 5.64%. The NPV was recorded at 94.74%, while precision was 95.34%, recall was 94.36%, and the F1 score was 94.85%. These results indicate that the model was highly effective in distinguishing Glioma cases, maintaining both high precision and recall, and minimizing false positives and false negatives [1]. **In the case of Meningioma**, the model reported a slightly lower accuracy of 94.28%. However, it showed a very low specificity of 97.46% and an FNR of 17.24%. The NPV was 95.34%, precision was 90%, recall was 83.76%, and the F1 score was 86.23%. The unusually low specificity and NPV suggest challenges in accurately identifying non-Meningioma cases, potentially due to class imbalance or overlapping features, despite having acceptable accuracy. **For Pituitary tumors**, the model achieved the highest accuracy at 96.77%, with a specificity of 96.10% and an FNR of 1.67%. It demonstrated an outstanding NPV of 99.27%, along with a precision of 91.47%, a recall of 98.33%, and an F1 score of 94.78%. These metrics illustrate excellent sensitivity and reliability in detecting Pituitary tumors, with very few false negatives.

In the fourth experiment, we validated the ViT-PCA-RF model using an external dataset from Figshare. We employed five DL models—ViT, VGG16, ResNet50, DenseNet201, and Xception—for feature extraction. We then applied PCA to reduce the dimensionality of the features and utilized the RF algorithm for classification.

The primary aim was to evaluate the feature extraction effectiveness of the ViT model in comparison to VGG16, ResNet50, DenseNet201, and Xception. The Figshare dataset consisted of three main classes: Meningioma, Pituitary, and Glioma. Results from this experiment, including hybrid models like ViT-PCA-RF, VGG16-PCA-RF, ResNet50-PCA-RF, DenseNet201-PCA-RF, and Xception-PCA-RF, are displayed in Table 23. This table illustrates the average performance metrics of the five hybrid models in the multi-classification task on the test set of the Figshare dataset. The average accuracies recorded were ViT-PCA-RF: 99%, VGG16-PCA-RF: 88.39%, ResNet50-PCA-RF: 90.71%, DenseNet201-PCA-RF: 92.37%, Xception-PCA-RF: 89.72%. As a result, the ViT-PCA-RF model demonstrated the highest accuracy among all models.

**ViT-PCA-RF** achieved the highest overall performance with specificity of 99.4% and recall of 98.1%. Its FNR was the lowest at 2.1%, while both precision and F1 score were at 98.1%, indicating a highly balanced and reliable classification. The high specificity and NPV of 99.4% suggested that this model effectively identified negative cases, minimizing false positives and false negatives.

**DenseNet201-PCA-RF** followed with specificity of 94.01% and recall of 85.77%. Its FNR was 14.23%, exhibiting a relatively lower rate of false negatives compared to most models, except for ViT-PCA-RF. Precision was at 86.88%, and the F1 score was 86.02%, reflecting a solid balance between precision and recall, though it underperformed relative to ViT-PCA-RF.

**ResNet50-PCA-RF** achieved specificity of 91.98%, recall of 80.98%, and an F1 score of 82.15%. Its FNR was 19.02%, indicating a greater tendency to miss positive cases. Precision was at 86.30%, slightly better than DenseNet201-PCA-RF, although the model’s balance leaned more towards higher precision at the expense of recall.

**Xception-PCA-RF** recorded specificity of 91.00%. Its recall was 78.57%, with an F1 score of 79.95% and an FNR of 21.43%. The model’s lower recall and higher FNR suggested more frequent misclassification of positive instances, despite a reasonable precision of 85.43%.

Lastly, **VGG16-PCA-RF** exhibited the lowest performance among the five models, with specificity of 89.70%, and recall of 75.82%. It had the highest FNR at 24.18% and the lowest F1 score at 77.02%, indicating struggles in correctly identifying positive cases and maintaining a balance between precision (84.20%) and recall (75.82%) [5].

In summary, ViT-PCA-RF outperformed the other models across all metrics, demonstrating superior accuracy, sensitivity, and specificity. DenseNet201-PCA-RF was the second-best model, while VGG16-PCA-RF lagged in all aspects. These results are consistent with prior findings suggesting that ViT can achieve state-of-the-art performance when combined with PCA and RF.

### 4.5. Comparing the Results with the Recent Literature

The hybrid ViT-PCA-RF model demonstrated exceptional accuracy of 99% in multi-classification tasks, showcasing its strong performance in the assigned objective. As a result, this fine-tuned hybrid model achieved superior outcomes compared to recent techniques outlined in Table 24. Additionally, the hybrid model is poised to assist healthcare professionals in the early detection of brain tumors, thereby reducing both diagnosis time and costs, while also aiding in curtailing the spread of such tumors. The ViT-PCA-RF model has proven its ability to surpass other contemporary classifiers through rigorous parameter tuning.

## 5. Conclusions

In this paper, a novel hybrid model called ViT-PCA-RF has been introduced to detect brain tumors effectively. This model utilized ViT for feature extraction, PCA for dimension reduction, and RF for tumor classification. The main goal is to quickly identify brain tumors, leading to improved patient outcomes and a more streamlined diagnostic process that reduces time and costs. Two experiments were carried out. The first experiment involved using ViT for feature extraction, PCA for dimension adjustment, and RF, DT, XGB, and SVM for classification to distinguish between different types of brain tumors. The second experiment categorized brain tumors using DL models such as ViT, VGG16, ResNet50, DenseNet201, and Xception for feature extraction. PCA was then applied for dimension reduction and RF for classification to assess ViT’s extraction effectiveness compared to other models. The models were evaluated using the BTM dataset to categorize brain tumors. The dataset underwent resizing and normalization and was split into training (81.33%—5712 CT images) and testing sets (18.67%—1311 CT images). The innovative ViT-PCA-RF model outperformed traditional classifiers, demonstrating the effectiveness of this approach in accurately identifying brain tumors. The proposed model achieved high accuracy, specificity, FNR, NPV, precision, recall, and F1 score for multi-classification at 99%, 99.4%, 2.1%, 99.4%, 98.1%, 98.1%, and 98.1%, respectively, showcasing superior performance compared to recent models. However, the running time remains a limitation of the system. Plans include extending the forecasting period of the model through further enhancements and component identification.

## Figures and Tables

**Table 1:** The classes’ distribution of the BTM datasets.

Dataset	Class	Image Count
BTM	Normal	2000
	Meningioma	1645
	Pituitary	1757
	Glioma	1621
Total	7023	

**Table 2:** The classes’ distribution of the training set.

Dataset	Class	Image Count
BTM	Normal	1595
	Meningioma	1339
	Pituitary	1457
	Glioma	1321
Total	5712	

**Table 3:** The classes’ distribution of the test set.

Dataset	Class	Image Count
BTM	Normal	405
	Meningioma	306
	Pituitary	300
	Glioma	300
Total	1311	

**Table 4:** The ViT’s structure for feature extraction.

Layer	Output Shape	Number of Parameters
Input Layer	(224, 224, 3)	0
ViT-B16	(None, 768)	85,798,656
Flatten_1	(None, 768)	0
Dense (Feature layer)	(None, 64)	49,216
Dense2	(None, 32)	2080
Dense3	(None, 4)	132

**Table 5:** The hyperparameters utilized in the four experiments.

Parameter	Value
img_size	224 × 224
Number of epochs	5
Batch size (BS)	16
Activation	softmax
Optimizer	AdamW
Initial learning rate	0.0001

**Table 6:** The processing time of the DL models as features extractor.

Model	Training Time	Testing Time	Feature Extraction Time for Training	Feature Extraction Time for Test	No. Params	Trainable Params	Non-Trainable Params	Accu. After 5 Epoch%
VIT	12.0 min, 42.36 s	10.53 s	46.22 s	10.33 s	85,850,084	85,850,084	0	94.508
VGG16	1.0 min, 16.65 s	2.97 s	10.89 s	2.61 s	14,714,688	14,714,688	0	43.326
ResNet50	1.0 min, 9.95 s	3.49 s	12.75 s	2.33 s	23,587,712	23,534,592	53,120	30.892
DenseNet201	1.0 min, 51.24 s	6.77 s	19.92 s	5.82 s	18,321,984	18,092,928	229,056	77.498
Xception	1.0 min, 28.92 s	4.01 s	17.45 s	3.19 s	20,861,480	20,806,952	54,528	73.837

**Table 7:** The processing time of the hybrid models.

Model	Training Time	Testing Time
ViT-PCA-RF	2.21 s	0.02 s
VGG16-PCA-RF	2.78 s	0.03 s
ResNet50-PCA-RF	3.02 s	0.03 s
DenseNet201-PCA-RF	2.75 s	0.03 s
Xception-PCA-RF	2.76 s	0.03 s

**Table 8:** The outcomes of the four hybrid models when assessed using test set of the BTM dataset.

Model	Accuracy (%)	Specificity (%)	FNR (%)	NPV (%)	Precision (%)	Recall (%)	F1 Score (%)
ViT-PCA-RF	99.0	99.4	2.1	99.4	98.1	98.1	98.1
ViT-PCA-DT	98.2	98.9	3.8	98.9	96.5	96.5	96.5
ViT-PCA-XGB	98.9	99.2	2.5	99.3	97.7	97.7	97.7
ViT-PCA-SVM	98.8	99.2	2.5	99.2	97.6	97.6	97.6

**Table 9:** The outcomes of ViT-PCA-RF when assessed using test set of the BTM dataset.

**ViT-PCA-RF**	**Class**	**Accuracy (%)**	**Specificity (%)**	**FNR (%)**	**NPV (%)**	**Precision (%)**	**Recall (%)**	**F1 Score (%)**
	Glioma	98.47	99.60	5.33	98.44	98.61	94.67	96.60
	Meningioma	98.55	98.81	2.29	99.30	96.14	97.71	96.92
	Normal	99.85	99.78	0.00	100	99.51	100	99.75
	Pituitary	99.31	99.31	0.67	99.80	97.70	99.33	98.51
	**Average**	**99**	**99.4**	**2.1**	**99.4**	**98.1**	**98.1**	**98.1**
	**Standard Deviation**	**0.6**	**0.4**	**2.1**	**0.6**	**1.2**	**2.1**	**1.3**

**Table 10:** The outcomes of ViT-PCA-DT when assessed using test set of the BTM dataset.

**ViT-PCA-DT**	**Class**	**Accuracy (%)**	**Specificity (%)**	**FNR (%)**	**NPV (%)**	**Precision (%)**	**Recall (%)**	**F1 Score (%)**
	Glioma	97.3	99.2	9.3	97.3	97.1	90.7	93.8
	Meningioma	97.2	97.4	3.6	98.9	91.9	96.4	94.1
	Normal	99.8	99.7	0.0	100	99.3	100	99.6
	Pituitary	98.8	99.1	2.3	99.3	97.0	97.7	97.3
	**Average**	**98.2**	**98.9**	**3.8**	**98.9**	**96.5**	**96.5**	**96.5**
	**Standard Deviation**	**1.1**	**0.9**	**3.4**	**1.0**	**2.7**	**3.4**	**2.4**

**Table 11:** The outcomes of ViT-PCA-XGB when assessed using the test set of the BTM dataset.

**ViT-PCA-XGB**	**Class**	**Accuracy (%)**	**Specificity (%)**	**FNR (%)**	**NPV (%)**	**Precision (%)**	**Recall (%)**	**F1 Score (%)**
	Glioma	98.2	99.4	5.7	98.3	97.9	94.3	96.1
	Meningioma	98.1	98.5	3.3	99.0	95.2	96.7	95.9
	Normal	99.8	99.7	0.0	100	99.3	100	99.6
	Pituitary	99.3	99.4	1.0	99.7	98.0	99.0	98.5
	**Average**	**98.9**	**99.2**	**2.5**	**99.3**	**97.7**	**97.7**	**97.7**
	**Standard Deviation**	**0.7**	**0.4**	**2.2**	**0.6**	**1.5**	**2.2**	**1.6**

**Table 12:** The outcomes of ViT-PCA-SVM when assessed using test set of the BTM dataset.

**ViT-PCA-SVM**	**Class**	**Accuracy (%)**	**Specificity (%)**	**FNR (%)**	**NPV (%)**	**Precision (%)**	**Recall (%)**	**F1 Score (%)**
	Glioma	98.4	99.2	4.3	98.7	97.3	95.7	96.5
	Meningioma	98.2	98.8	3.6	98.9	96.1	96.4	96.2
	Normal	99.2	99.3	1.2	99.4	98.5	98.8	98.6
	Pituitary	99.5	99.5	0.7	99.8	98.3	99.3	98.8
	**Average**	**98.8**	**99.2**	**2.5**	**99.2**	**97.6**	**97.6**	**97.6**
	**Standard Deviation**	**0.5**	**0.3**	**1.5**	**0.4**	**1.0**	**1.5**	**1.2**

**Table 13:** The accuracies of the hybrid models when assessed using the test set of the BTM dataset.

Model	Accuracy (%)	Specificity (%)	FNR (%)	NPV (%)	Precision (%)	Recall (%)	F1 Score (%)
ViT-PCA-RF	99.0	99.4	2.1	99.4	98.1	98.1	98.1
VGG16-PCA-RF	97.0	98.0	6.5	98.1	94.0	94.0	93.9
ResNet50-PCA-RF	95.7	97.2	9.4	97.2	91.2	91.3	91.2
DenseNet201-PCA-RF	96.2	97.5	8.3	97.6	93.0	92.4	92.3
Xception-PCA-RF	95.3	96.9	10.2	97.0	90.6	90.5	90.5

**Table 14:** The outcomes of VGG16-PCA-RF when assessed using the test set of the BTM dataset.

**VGG16-PCA-RF**	**Class**	**Accuracy (%)**	**Specificity (%)**	**FNR (%)**	**NPV (%)**	**Precision (%)**	**Recall (%)**	**F1 Score (%)**
	Glioma	95.3	98.4	15.3	95.6	94.1	84.7	89.1
	Meningioma	94.8	96.1	9.5	97.1	87.7	90.5	89.1
	Normal	99.6	99.4	0	100	98.8	100	99.4
	Pituitary	98.2	98.1	1.3	99.6	94.0	98.7	96.3
	**Average**	**97**	**98**	**6.5**	**98.1**	**94**	**94**	**93.9**
	**Standard Deviation**	**2.0**	**1.2**	**6.2**	**1.8**	**4.0**	**6.2**	**4.5**

**Table 15:** The outcomes of ResNet50-PCA-RF when assessed using the test set of the BTM dataset.

**ResNet50-PCA-RF**	**Class**	**Accuracy (%)**	**Specificity (%)**	**FNR (%)**	**NPV (%)**	**Precision (%)**	**Recall (%)**	**F1 Score (%)**
	Glioma	93	96.7	19.7	94.3	88	80.3	84
	Meningioma	92.1	94.6	16.3	95	82.6	83.7	83.1
	Normal	99.5	99.3	0	100	98.5	100	99.3
	Pituitary	98	97.9	1.7	99.5	93.4	98.3	95.8
	**Average**	**95.7**	**97.2**	**9.4**	**97.2**	**91.2**	**91.3**	**91.2**
	**Standard Deviation**	**3.2**	**1.7**	**8.7**	**2.6**	**6.0**	**8.7**	**7.1**

**Table 16:** The outcomes of DenseNet201-PCA-RF when assessed using the test set of the BTM dataset.

**DenseNet201-PCA-RF**	**Class**	**Accuracy (%)**	**Specificity (%)**	**FNR (%)**	**NPV (%)**	**Precision (%)**	**Recall (%)**	**F1 Score (%)**
	Glioma	94.2	99.1	22.3	93.7	96.3	77.7	86
	Meningioma	92.7	92.8	7.8	97.5	79.7	92.2	85.5
	Normal	99.9	100	0.2	99.9	100	99.8	99.9
	Pituitary	97.9	98.1	2.7	99.2	93.9	97.3	95.6
	**Average**	**96.2**	**97.5**	**8.3**	**97.6**	**93**	**92.4**	**92.3**
	**Standard Deviation**	**2.9**	**2.8**	**8.6**	**2.4**	**7.7**	**8.6**	**6.2**

**Table 17:** The outcomes of Xception-PCA-RF when assessed using the test set of the BTM dataset.

**Xception-PCA-RF**	**Class**	**Accuracy (%)**	**Specificity (%)**	**FNR (%)**	**NPV (%)**	**Precision (%)**	**Recall (%)**	**F1 Score (%)**
	Glioma	93.6	97.7	20.3	94.2	91.2	79.7	85.1
	Meningioma	91.6	94	16.3	95.0	81	83.7	82.3
	Normal	99.5	99.3	0.2	99.9	98.5	99.8	99.1
	Pituitary	96.4	96.5	4.0	98.8	89.2	96.0	92.5
	**Average**	**95.3**	**96.9**	**10.2**	**97.0**	**90.6**	**90.5**	**90.5**
	**Standard Deviation**	**3.0**	**1.9**	**8.3**	**2.4**	**6.2**	**8.3**	**6.6**

**Table 18:** The outcomes of the hybrid models when assessed using test set of the Figshare dataset.

Model	Accuracy (%)	Specificity (%)	FNR (%)	NPV (%)	Precision (%)	Recall (%)	F1 Score (%)
ViT-PCA-RF	95.85	96.54	8.10	97.05	93.94	91.90	92.74
ViT-PCA-DT	95.36	96.44	8.19	96.47	91.96	91.81	91.88
ViT-PCA-XGB	95.36	96.17	8.82	96.62	92.91	91.18	91.89
ViT-PCA-SVM	95.36	64.24	2.49	64.99	62.57	64.51	63.50

**Table 19:** The outcomes of ViT-PCA-RF when assessed using test set of the Figshare dataset.

**ViT-PCA-RF**	**Class**	**Accuracy (%)**	**Specificity (%)**	**FNR (%)**	**NPV (%)**	**Precision (%)**	**Recall (%)**	**F1 Score (%)**
	Glioma	95.52	93.72	2.56	97.49	93.60	97.44	95.4774
	Meningioma	95.02	98.73	18.39	95.11	94.67	81.61	87.6543
	Pituitary	97.01	97.16	3.33	98.56	93.55	96.67	95.082
	**Average**	95.85	96.54	8.10	97.05	93.94	91.90	92.74
	**Standard Deviation**	0.85	2.09	7.29	1.44	0.52	7.29	3.60

**Table 20:** The outcomes of ViT-PCA-DT when assessed using test set of the Figshare dataset.

**ViT-PCA-DT**	**Class**	**Accuracy (%)**	**Specificity (%)**	**FNR (%)**	**NPV (%)**	**Precision (%)**	**Recall (%)**	**F1 Score (%)**
	Glioma	95.52	95.65	4.62	95.65	95.38	95.38	95.38
	Meningioma	94.03	96.51	14.94	95.90	87.06	85.06	86.05
	Pituitary	96.52	97.16	5.00	97.86	93.44	95.00	94.21
	**Average**	95.36	96.44	8.19	96.47	91.96	91.81	91.88
	**Standard Deviation**	1.02	0.62	4.78	0.99	3.56	4.78	4.15

**Table 21:** The outcomes of ViT-PCA-XGB when assessed using the test set of the Figshare dataset.

**ViT-PCA-XGB**	**Class**	**Accuracy (%)**	**Specificity (%)**	**FNR (%)**	**NPV (%)**	**Precision (%)**	**Recall (%)**	**F1 Score (%)**
	Glioma	94.78	93.24	3.59	96.50	93.07	96.41	94.71
	Meningioma	94.28	98.10	19.54	94.79	92.11	80.46	85.89
	Pituitary	97.01	97.16	3.33	98.56	93.55	96.67	95.08
	**Average**	95.36	96.17	8.82	96.62	92.91	91.18	91.89
	**Standard Deviation**	1.19	2.11	7.58	1.54	0.60	7.58	4.25

**Table 22:** The outcomes of ViT-PCA-SVM when assessed using test set of the Figshare dataset.

**ViT-PCA-SVM**	**Class**	**Accuracy (%)**	**Specificity (%)**	**FNR (%)**	**NPV (%)**	**Precision (%)**	**Recall (%)**	**F1 Score (%)**
	Glioma	95.02	95.65	5.64	94.74	95.34	94.36	94.85
	Meningioma	94.28	97.46	17.24	95.34	90.00	82.76	86.23
	Pituitary	96.77	96.10	1.67	99.27	91.47	98.33	94.78
	**Average**	95.36	64.24	2.49	64.99	62.57	64.51	63.50
	**Standard Deviation**	1.04	44.74	2.31	45.32	43.64	45.06	44.29

**Table 23:** The accuracies of the hybrid models when assessed using the test set of the Figshare dataset.

Model	Accuracy (%)	Specificity (%)	FNR (%)	NPV (%)	Precision (%)	Recall (%)	F1 Score (%)
ViT-PCA-RF	99.0	99.4	2.1	99.4	98.1	98.1	98.1
VGG16-PCA-RF	88.39	89.70	24.18	92.47	84.20	75.82	77.02
ResNet50-PCA-RF	90.71	91.98	19.02	93.72	86.30	80.98	82.15
DenseNet201-PCA-RF	92.37	94.01	14.23	94.38	86.88	85.77	86.02
Xception-PCA-RF	89.72	91.00	21.43	93.31	85.43	78.57	79.95

**Table 24:** Comparison of the proposed model’s results with recent models’ results.

Reference	Methodology	Accuracy	Datasets
J. Wang et al. [19]	ViT	98.86%	BTM
Z. Rasheed et al. [20]	CLAHE, VGG16, ResNet50, VGG19, InceptionV3, and MobileNetV2	97.84	BTM
M. A. Gómez-Guzmán et al. [21]	Generic CNN, ResNet50, InceptionV3, InceptionResNetV2, Xception, MobileNetV2, and EfficientNet-B0	97.12	BTM
K. Abdul Hannan et al. [22]	CNN	92.13%	BMI-I, BMI-II, BMI-III, BTI, and BTS
A. Naseer et al. [23]	CNN	98.8%	BTM
Saeedi et al. [25]	a convolutional auto-encoder network and new 2D CNN	96.47%	BTM
Sarkar et al. [26]	LIME, CNN, SHAP	94.64%	BTM
E. Mohammed Senan et al. [27]	ResNet-18, AlexNet, and SVM	95.10%	BTM
**ViT-PCA-RF model**	**ViT, PCA, and RF**	**99%**	BTM

## Data Availability

The dataset used in this article is the BTM dataset. The benchmark dataset was sourced from https://www.kaggle.com/datasets/masoudnickparvar/brain-tumor-mri-dataset (Last accessed on 10 September 2022).
